# Editorial: Data-Driven Solutions for Smart Grids

**DOI:** 10.3389/fdata.2021.815686

**Published:** 2021-12-01

**Authors:** F. Milano, A. Vaccaro, M. Manana

**Affiliations:** ^1^ School of Electrical and Electronic Engineering, University College Dublin, Dublin, Ireland; ^2^ Department of Engineering, University of Sannio, Benevento, Italy; ^3^ Department of Electrical and Energy Engineering, University of Cantabria, Santander, Spain

**Keywords:** smart grid, optimization, energy policies and regulation, data-driven modelling, fault localisation and identification, state estimation (SE), data consistency and synchronization, virtual power plants (VPP)

This research topic deals with innovative data-driven solutions for smart grids. The integration of communication systems and smart metering in power system has led, in recent years, to capability to measure quantities and communicate measurements with a precision, a sampling rate and a bandwidth that were unimaginable just a few years ago. The potential for this amount of information is huge but the power system industry and community have not yet fully exploited such a capability.

In this complex data-rich, but information-limited domain, the data streaming generated by the pervasive grid sensors do not always provide smart grids operators with the necessary information to react to external disturbances in a timely manner. Even if fast computing algorithms are utilized to convert data into information, smart grid operators face the challenge of not having the full picture of the information context and, therefore, the obtained information cannot be deployed with a high degree of confidence.

To address this complex issue, the most promising research directions are oriented toward the conceptualization of improved information processing paradigms and smart decision support systems aimed at enhancing standard operating procedures, based on pre-defined grid conditions and static operating thresholds, with a set of interactive information services, which could promptly provide the right information at the right moment to the right decision maker. To effectively support the deployment of these services in modern smart grids it will be incumbent upon the scientific community to develop advanced techniques and algorithms for reliable power system data acquisition and processing, which should support semantics and content-based data extraction and integration from heterogeneous sensor networks.

This research topic contains four articles.

The paper *Optimal Balancing of Wind Parks with Virtual Power Plants* by Omelčenko and Manokhin addresses data-driven solutions in the context of optimization of virtual power plants. This work proposes the use of machine learning to process available data measurements. The goal is to balance the power production and at the same time maximize the revenue of a portfolio of power plants with different technologies (biogas, wind, batteries, etc.) considering uncertainty in both price and power production.

The paper *Supporting Regulatory Measures in the Context of Big Data Applications for Smart Grids* by Mladin discusses the policy and regulatory aspects. This paper focuses in particular on big data applications to the ongoing “energy transition” process built on higher renewable energy integration and digitalization, and discusses how this can help regulatory measures through societal acceptance and involvement.

The paper *Data Consistency for Data-Driven Smart Energy Assessment* by Chicco addresses the issue of data consistency and discusses data-versus model-based approaches. The latter is an emerging topic and a potential paradigm shift in power system analysis that have been traditionally based on model-based approaches. The paper illustrates how both approaches have advantages and drawbacks and discusses how to choose the right approach depending on the application and the properties of the available data.

Finally, the paper *Which Neural Network to Choose for Post-Fault Localization, Dynamic State Estimation, and Optimal Measurement Placement in Power Systems?* by Afonin and Chertkov discusses dynamic state estimation and the role of the choice of the appropriate neural network for the determination of optimal placement of PMUs for fault locations. The authors conclude that the answer to the question posed in the title of this work is not unique. It depends on which data is available, how much in detail the power system is to be estimated and how much extrapolation is considered acceptable.

Our goal in putting together this research topic is to provide a glance on the vast variety of aspects, spanning from market and policy to modelling and measurement as the power industry is going through an unprecedented energy transition and technological revolution. We hope that this topic will be of interest for regulators and practitioners as well as for the academic community.

